# Dual‐task disparities in gait and functional mobility among older adults with a fall‐related mild traumatic brain injury and people living with dementia

**DOI:** 10.1002/alz.087819

**Published:** 2025-01-09

**Authors:** Albert K Okrah, Stephen A Parada, Wanda D Jirau‐Rosaly, Max Bursey, Bradford Reynolds, Chijioke Ohamadike, Deborah A Jehu

**Affiliations:** ^1^ Augusta University, Augusta, GA USA

## Abstract

**Background:**

Mild traumatic brain injury (mTBI) increases dementia risk. Delays in diagnosis are common due to insensitive tools, prolonging symptoms and time to treatment. Dual‐task gait and functional mobility deficits are present post‐mTBI and in people living with dementia (PWD); however, it is unclear whether dual‐tasking can be used as a tool to differentiate between groups. This study aimed to 1) assess whether dual‐task mobility is more sensitive to detect differences between older adults with a mTBI relative to PWD than single‐task mobility; and 2) examine dual‐task cost between groups.

**Method:**

n = 3 community‐dwelling older adults with a fall‐related mTBI (Age:68.6 years, Montreal Cognitive Assessment (MOCA) = 23 points, 67% Female) and n = 25 PWD in residential care facilities (Age:82.1 years, MOCA = 10.2 points, 36% Female) wore 6 APDM inertial sensors and completed two usual pace walking trials (single‐task) with or without naming words (dual‐task). Participants also completed two trials of the timed‐up‐and‐go, which involved getting up from a chair, walking 3 m, turning around, walking back, and sitting down (single‐task) with or without completing a category task (dual‐task). Separate repeated measures analyses of variance were conducted for gait and the TUG.

**Result:**

There were no between‐group interactions (p>0.05). There was a Condition by Measure interaction for gait (F_(1.13,29.49)_ = 35.25, p<0.001, η_p_^2^ = 0.58) and functional mobility (F_(1.53,39.89)_ = 6.0, p<0.01, η_p_^2^ = 0.19). Dual‐tasking provoked slower gait speed (dual‐task:0.36 m/s; single‐task:0.54 m/s), greater double limb support (dual‐task:38.0%; single‐task:31.8%), lower midswing elevation (dual‐task:0.91cm; single‐task:1.28cm), and shorter stride length (dual‐task:0.55m; single‐task:0.71m;p<0.001) than single‐tasking. The dual‐task TUG provoked longer time to completion (dual‐task:33.2s; single‐task:22.2s), smaller turn angle (dual‐task:120.7°; single‐task:140.2°), and slower turn velocity (dual‐task:80.3m/s; single‐task:94.1m/s) than the single‐task TUG.

**Conclusion:**

There were no between‐group differences in single‐ or dual‐task mobility; however, these findings should be interpreted with caution as we have only tested n = 3 participants in the mTBI group, and data collection is ongoing. Both groups experienced some level of cognitive impairment, highlighting that the deficits observed in dual‐ relative to single‐task mobility were likely a manifestation of increased attention demand. Further work is underway to determine whether dual‐tasking may be a sensitive measure to screen and monitor mobility impairments in these populations.

